# Clinical Report on Pneumonia

**Published:** 1859-08

**Authors:** 


### EXTRACTS.

### CLINICAL REPORT ON PNEUMONIA.

Dr. Austin Flint has published (*Buffalo Medical Journal*, May, 1859) an interesting clinical report on cases of pneumonia, observed by him at the New Orleans Charity Hospital, 1858 and 1859.

We subjoin his conclusions and remarks.

Of the fifteen cases, in all the pneumonia was limited to a single lobe. In one instance two lobes were successively attacked (cases 3 and 4), recovery from the first attack having taken place before the second occurred. This was probably true in another instance (case 9.) The lower lobe was the seat of the disease in all save two cases. In one of these (case 4), they had been previously affected, and this was probably true of the other instance (case 9).

Of the thirteen cases in which the lower lobe was affected, the disease was seated in the right side in seven, and in the left side in six.

The pneumonia was primary in twelve of the fifteen cases. Of the three instances in which it was developed secondary, it occurred as a complication of typhoid fever in two, and as an intercurrent affection in pulmonary tubercolosis in one instance.

In the twelve primary cases, the disease was uncomplicated in all but three instances. Delirium tremens was developed as a complication in ten cases; and in one instance, pleuritis with considerable liquid effusion coexisted. More or less pleuritis is almost uniformly present in cases of pneumonia, but it is certainly rare for the plueritis, under these circumstances, to be attended by much effusion.

The ages of the patients varied between 20 and 45, the mean age being about 30. In nearly every instance the patients were robust and in good health when attacked with the disease. Several were intemperate, and several were subject to attacks of intermittent fever.

Exclusive of three cases remaining in hospital at the end of my term of service (two of these remaining with other affections), and of the case which ended fatally the length of stay in hospital varied from six to twenty-two days; the mean period being a fraction over thirteen days.

A fatal result occurred in one case only. In this case, as already stated, the patient had been discharged a few days before, convalescent from an attack of pneumonia affecting the lower lobe of the right lung. He resumed his habits of intemperance directly he was discharged, and was seized a second time with pneumonia, now affecting the upper lobe of the left lung. He walked to the hospital, a distance of three miles, after being attacked, and died by asthenia, fifteen hours after his admission, without coming under my observation.

Convalescence was rapid in all instances, save one in which the pneumonia occurred as a complication of typhoid fever. Exclusive of this instance, the resolution of the affected lung, as shown by the disappearance of the physical signs denoting solidification, was rapid. The resolution was in all instances complete, slight relative dulness on percussion only remaining, which, as is well known, persists for some time after recovery from pneumonia. In all instances the symptoms of the disease, viz., cough, expectoration, etc., completely disappeared. In no instance were the patients left enfeebled, but at the time of discharge they all reported themselves able to return to their avocations, and the majority were day laborers.

The treatment pursued in these cases, as the reader has doubtless remarked, was not complex. The sulphate of quinia was given in tolerably full doses, in the cases in which intermittent fever had previously existed. This remedy was not prescribed with special reference to the pneumonia, but in order to forestall the development of intermittent fever as a complication. Quinia has been extolled as a valuable remedy in pneumonia, irrespective of the liability of the disease to become complicated with intermittent fever if the patient reside in a malarious section, and, especially, if he has been subject to attacks of the latter affection. However this may be, it is certain that the remedy, given in full doses, does not exert an unfavorable influence on the progress of the pneumonia, It is not contra-indicated by this affection. To prevent the development of intermittent fever, and if developed, to arrest the paroxysms as speedily as possible, are important objects of treatment in certain cases of the disease under consideration. The combination of the two affections often places the patient in imminent danger, when with either affection, singly, his condition would not be serious. Happily, under these circumstances, we possess a special remedy by which we may expect to control one of the affections. This, then, is the leading indication, and the life of the patient may depend on its being promptly and effectively fulfilled. The practitioner who gives precedence to indications derived from the pneumonia, under these circumstances, or who so divides his attention as to fulfil incompletely the leading indication, commits an error which may be fatal to the patient. As a rule, it is well to recognize, at the commencement, a liability to this complication, and to forestall its occurrence, especially when the protective remedy, to say the least, does not interfere with the favorable progress of the pneumonia. • •

Opium entered more or less into the treatment of these cases. My observations have led me to regard this as a valuable remedy in pneumonia. As a palliative of pain, it fulfils an important indication; for pain, in addition to the suffering which it occassions, determines an afflux of blood to the painful part. This fact is illustrated in neuralgia affecting the supra-obital nerve. During the paroxysm of pain, the eye becomes injected, and the redness rapidly disappears after the pain has been relieved by an opiate. But there is reason to believe that opium exerts a salutary effect beyond the relief of pain. I have repeatedly noted marked diminution in the frequency of the pulse and respirations, in cases of pneumonia, a few hours after the patient had taken full doses of opium. It appears to lessen the perturbatory effects in the economy of the local inflammation, if, indeed, it does not diminish the intensity of the inflammatory action. Some practitioners are deterred from the use of this remedy in pneumonia, by the idea that it interferes with the removal, by expectoration, of the exuded products of inflammation. But how often do we observe the rapid disappearance of the solidification in pneumonia, with slight expectoration, or none whatever! The intra-vesicular exudation is, for the most part, absorbed, not expectorated, a fact not strange when the structure of the cells, and the facility of endosmosis in this situation are considered. Interference with expectoration, therefore if true with respect to the employment of opium in pneumonia, is not a valid objection. The merits of the remedy, however, in this application, must rest on clinical experience; and I cannot but think that this paper will be of some utility, should it serve to remove from the minds of some readers, groundless apprehensions with respect to the free use of opium in pneumonia.

Alcoholic stimulants were employed to a greater or less extent, in the treatment of these cases, and in all the cases the patients were placed on a nutritious diet. The abstract notion that stimulants and sustaining food are inconsistant with the treatment due to local inflammation, is a remnant of Broussaism which still exerts considerable influence on medical practice. It suffices to destroy this notion, to bear in mind the significant injunction of Chomel, viz., *not to treat diseases, but to treat patients affected with disease.* With regard to stimulants, it may be stated, as a rule, that they are indicated in cases of local inflammation, whenever the patient in health is addicted to their use. They cannot with safety be withheld, under these circumstances, if the local inflammation involve, from its seat or intensity, danger to life. But without reference to previous habits, the use of stimulants in the treatment of pneumonia, and of other local inflammations, is important in proportion as it becomes an indication to support the powers of the system. It is, perhaps, an error in medical practice as common and as serious as any, to overlook or depreciate this indication in the management of local diseases. Practically, the existence of this indication and its urgency, in individual cases, may be brought clearly and forcibly before the mind by proposing at the bedside the following questions: Is the patient in danger of death? If death occur, will it take place by asthenia? How imminent is the danger of death by asthenia? Whenever, in the management of disease, the physician has reason to fear that he may lose his patient by asthenia, and in proportion to the tendency to a fatal result by that mode of dying, alcholic stimulants, as a rule, form an important part of the measures indicated, on the same ground that they enter into the treatment of fevers. And this remark applies equally to nutritious diet, viz., the animal essences, etc. Cases of pneumonia occur in which patients are to be saved by pursuing, boldly and perseveringly, the supporting treatment precisely as in cases of low typhus or typhoid fever. And the indications of this plan of treatment may be present early, as well as late in the progress of the disease. The union of delirium tremens, with pneumonia, for example, often places the patient in imminent danger of death by asthenia. The free use of stimulants and concentrated nutriment, is requisite to carry him safely through the perils of this combination. Two of the cases included in the present collection (Nos. 13 and 14) illustrate this remark.

But even when the danger to life is not great, it is an object to support the system with a view to a speedy and rapid resolution of the inflammation. Alcoholic stimulants may not be required for this object, but it will be promoted by a nutritious diet. The fear of feeding the disease by feeding the patient, is a vulgar notion not warranted by clinical observation. I do not hesitate to allow patients to take food as nutritious in quality and as freely as they desire. The late Dr. Graves was so impressed with the importance of supplying the system with nourishment in febrile diseases, that he desired to be placed as an epitaph on his tomb, "he fed fevers!" He might have extended the circle of diseases in which feeding is important. It is hardly less important, in some instances, speaking metaphorically, to feed pneumonias, than to feed fevers, and, indeed, feeding, as an essential element of supporting treatment, is important in any disease when it is an indication to obviate the tendency to death by asthenia.

With regard to the time when an active supporting plan of treatment (stimulants and concentrated nutriment) is indicated, and the extent to which it is to be carried, I will make but a single additional remark. If there be room for doubt on these points, it is better to err by commencing too soon, and pushing it too far, than by delay and insufficiency; for while it is easy to discontinue or diminish supporting measures before much, if any, actual harm is done, time which has been lost cannot be recovered.

Quinia, opium, alcoholic stimulants and nutritious diet, constituted the treatment in the cases embraced in this report. The grounds for the non-employment of certain therapeutical measures, in these cases, are now to be considered. Blood-letting, general and local, tartar emetic, the veratrum viride, mercury, cathartics, blisters, and other modes of counter-irritation are employed, to a greater or less extent, in the treatment of pneumonia. None of these remedies, however, entered into the treatment of these cases. This fact, without explanation, might lead to erroneous inferences as regards the views of the writer.

Blood-letting in pneumonia has, of late, been much discussed. I have contributed my mite towards this discussion,* and do not propose to enter upon it in this paper. I will simply remark, that, in my opinion, it is an error to assume blood-letting to be never useful in pneumonia, albeit it is a far greater error to advocate the indiscriminate employment of this spoliative measure in that affection. The propriety of blood-letting, however, is rarely a question in hospital cases of pneumonia. In the great majority of instances when patients are admitted into an hospital, the disease has advanced to the second stage; one or more lobes

* Report on Blood-letting in Pneumonia, *Buffalo Medical Journal*, vol. xi. 1856. Page 288.

are solidified, and, under these circumstances, the abstraction of blood will, in general, only tend to retard resolution. Had these cases been under observation from the commencement of the disease, it is possible that blood-letting might have been indicated in some instances, not with a view to arrest the inflammatory action, nor to limit its extent, nor to lessen the amount of exuded products; but to diminish the intensity of symptomatic febrile movement, relieving the heart of over-accumulation of blood in its cavities, and consequent over-tasking of its powers. With this limited view of blood-letting, making due allowance for its evils, it is indicated in only a small proportion even of the cases of pneumonia which come under observation at the onset of the disease.

The tartar emetic and veratrum viride undoubtedly control, in a marked degree, the frequency of the heart's action, and, so far, diminish, for the time, symptomatic febrile movement. It remains, however, to be determined, to what extent this effect is important with reference to the inflammatory process and resolution. We can at this day understand how Laennec was deceived in attributing the rapid removal of the exudation in pneumonia to the influence of tartar emetic; for it had not then been observed, as it has been since his day, that the exudation disappears, in some instances, quite as rapidly without any treatment. These cardiac sedatives, if useful when the symptomatic febrile movement is intense, are certainly not indicated when the action of the heart is but slightly or moderately increased, as in a pretty large proportion of the cases of pneumonia in which the affection is limited to a single lobe, especially after exudation has taken place.

The action of mercury has been considered as useful in two ways in pneumonia, viz., limiting the amount of exudation and promoting its absorption. With reference to the first of these supposed effects, the exudation in pneumonia generally takes place, to its fullest extent, before mercurialization can be produced. With reference to the second effect, the exudation is absorbed, in favorable cases, quite rapidly without mercurialization. Without, therefore, presuming to deny altogether the so-called anti-plastic and the sorbefacient powers of mercury, I believe that it is a remedy generally uncalled for in the treatment of pneumonia. The occurrence of salivation at the time of convalescence, is, to say the least, an inconvenience which, if not necessary, it is desirable to avoid. Having for many years relinquished the use of this remedy with reference to its special or constitutional effects in treating this disease, I am satisfied that it may with safety and advantage be dispensed with.

Cathartics, as a means of depletion during the early stage of pneumonia, may be indicated. If adequate to fulfil the object of depletion, they are certainly to be preferred to blood-letting, since they do not involve an expenditure of the organized constituents of the blood, and are therefore not spoliative. Except for this purpose, it may fairly be doubted whether they are called for in pneumonia. They conflict with supporting measures when these are indicated. That they are not important with reference to the absorption of the exudation, is sufficiently illustrated by the cases reported in this paper. In one of the cases (No. 15) considerable liquid effusion, together with the solidifying deposit, disappeared in a few days, although, inadvertently, the bowels had been permitted to remain constipated for a week.

Blisters and other modes of active counter-irritation, when employed in pneumonia, are, of course, considered as acting usefully by way of revulsion. I suppose that no judicious physician attributes to them this action during the first stage of the disease. To employ them in this stage is to add a certain amount of cutaneous inflammation to the existing pulmonary inflammation. After solidification has taken place, all the results to which the inflammation may be expected to give rise, have already occurred. Revulsion, then, where it is attainable, ceases to be an indication. Is it imagined that a traumatic inflammation of the skin diminishes the afflux of blood to the affected lung? But the affected lung in the second stage of pneumonia is already anæmic! The inconvenience occasioned by blisters to the patient is considerable; they are also a source of inconvenience to the practitioner, by interfering with the daily examinations of the chest in order to determine the physical condition of the affected lung. These remarks, of course, do not apply to mild revulsive applications, such as sinapisms, and to fomentations which doubtless possess a certain value in the treatment of pneumonia.

The several therapeutical measures, thus, which have been enumerated, as not entering into the treatment of the cases of pneumonia now reported, were not employed, because, with reference to some of them, the disease did not come under observation sufficiently early to find the indications for their use, or, the indications existing in a certain proportion of cases only, they did not happen to be present in these cases; and with reference to other of the measures mentioned, their value and propriety in the disease under consideration are, to say the least, doubtful. As the modern conservative surgeon rejects the doctrine contained in the old aphorism, *melius anceps remedium*

*quam nullum;* so the conservative physician should refuse to resort to remedies which are either unnecessary or of questionable utility, except in the cases in which a deviation from this rule is warranted for experimental observation. I cannot but think that the practice of medicine would gain much if practitioners, instead of deliberating at the bedside as to whether this or that potent remedy is called for, were oftener in the habit of propounding to themselves this inquiry: Are there present any clear indications for interference?

The last remark suggests a point of fundamental importance in the treatment of this, as well as other acute diseases, viz., its intrinsic tendency as regards termination. Does pneumonia, in itself, tend to destroy life? We might answer this question by citing the results as reported by different practitioners, the disease being treated by different and sometimes quite opposite measures. But the clinical study of the disease uninfluenced by medication, pursued of late years on an extended scale, enables us to answer the question more directly. The results reported by Dr. Dietl, and others, in which, in a large number of cases, no active remedies were employed, show that pneumonia, limited to a single lobe, and uncomplicated, ends in recovery in the vast majority of instances. This statement is, of course, exclusive of the epidemic forms of the disease; but it is probable that the fatality of epidemic pneumonia depends generally on some important complication, or associated affection. A series of cases of sporadic, uncomplicated pneumonia, therefore, ending favorably, under a certain plan of treatment, does not afford adequate evidence that the success was due to the treatment. It would be a fairer conclusion to impute the success to the intrinsic tendency of the disease to recovery. On the other hand, a series of cases in which the fatal cases were not few, would justify, at least, a suspicion that the want of success might be due to the treatment. It would, however, be an error to suppose that because pneumonia, under favorable hygienic circumstances, generally ends in recovery without medicinal treatment, that medication is consequently never called for. Better far, indeed, no treatment than injudicious interference; but judicious treatment may nevertheless save some lives which would be lost under the expectant plan. Moreover, there are objects of treatment in disease, in addition to recovery. Relief of distressing symptoms during the progress of disease; a cure *cito et jucunde* as well as sure; a convalescence rapid, and a recovery complete, leaving the powers of the body not permanently impaired—these are important ends to be kept in view in medical practice. The natural history of a disease, and its intrinsic tendency to life or death, constitute the true point of departure for the study of therapeutics in relation to that disease; but medical art should not be content with being able to state the chances of recovery, even when the chances are vastly in favor of this termination.—*American Journal of the Medical Sciences.*

